# The Solo Anterior Cruciate Ligament Technique: Using an Articulated Arm Holder for Anterior Cruciate Ligament Reconstruction Without a Surgical Assistant

**DOI:** 10.1016/j.eats.2024.103196

**Published:** 2024-08-22

**Authors:** Rafael A. Buerba, Jorge Garavito, Nickolas F. Fretes

**Affiliations:** a Banner University Sports Medicine Center, Department of Orthopaedic Surgery, University of Arizona College of Medicine-Phoenix, Phoenix, Arizona, U.S.A.; b The Warren Alpert Medical School of Brown University, Providence, Rhode Island, U.S.A.; c Martin Luther King Jr. Medical Center/Charles R. Drew University of Medicine and Science, Department of Orthopaedic Surgery, Los Angeles, California, U.S.A.

## Abstract

The Federal No Surprises Act was passed to avoid surprise out-of-network billing to patients, particularly after surgical procedures from independent third parties hired as assistants. This has resulted in a scarcity of “for-hire” surgical first assistants. Anterior cruciate ligament (ACL) surgery is challenging to perform without an assistant. During arthroscopy, the leg must be manipulated simultaneously while performing surgery. This usually requires multiple hands and can be physically demanding. This Technical Note will describe the use of an articulated arm holder adapted to hold and manipulate a leg during ACL surgery without an assistant as well as the use of a self-retractor to aid in quadriceps tendon graft harvest and repair when performing a solo quadriceps tendon autograft ACL reconstruction.

Anterior cruciate ligament (ACL) surgery is particularly challenging to perform without an assistant. During arthroscopy, the leg must be manipulated simultaneously while performing surgery, which requires multiple hands and can be physically demanding. Throughout orthopaedic training, most trainees learn to perform ACL surgery with an assistant. However, once in practice, early-career surgeons may find it difficult to obtain an experienced ACL surgical assistant.

Obtaining surgical assistance is becoming increasingly challenging in today’s health care environment. A principal contributing factor is the Federal No Surprises Act, which helps minimize surprise out-of-network billing to patients by prohibiting the hiring of independent third-party surgical assistants. This Act has required many of the “for-hire” surgical assistants to establish contracts with hospitals and medical groups to provide their services, restricting their overall availability, causing a scarcity.^1^^,^^2^

In addition, many early-career orthopaedic surgeons do not have access to physician assistants until they meet productivity thresholds. Nonacademic surgeons and junior attendings also may not have readily access to fellows, residents, and medical students for assistance. Finally, the COVID-19 pandemic decreased the number of health care personnel in the elective surgery realm, making it difficult for surgeons to have 2 scrub technicians with arthroscopy experience.^3^ This can be frustrating for an early-career surgeon, as it is difficult to increase productivity without the appropriate surgical assistance. This Technical Note proposes a solution to safely perform ACL surgery without an assistant by using an articulated arm holder adapted to hold a leg. It also describes the use of a self-retractor to aid in solo quadriceps tendon graft harvest and repair.

## Surgical Technique

**Fig 1:**
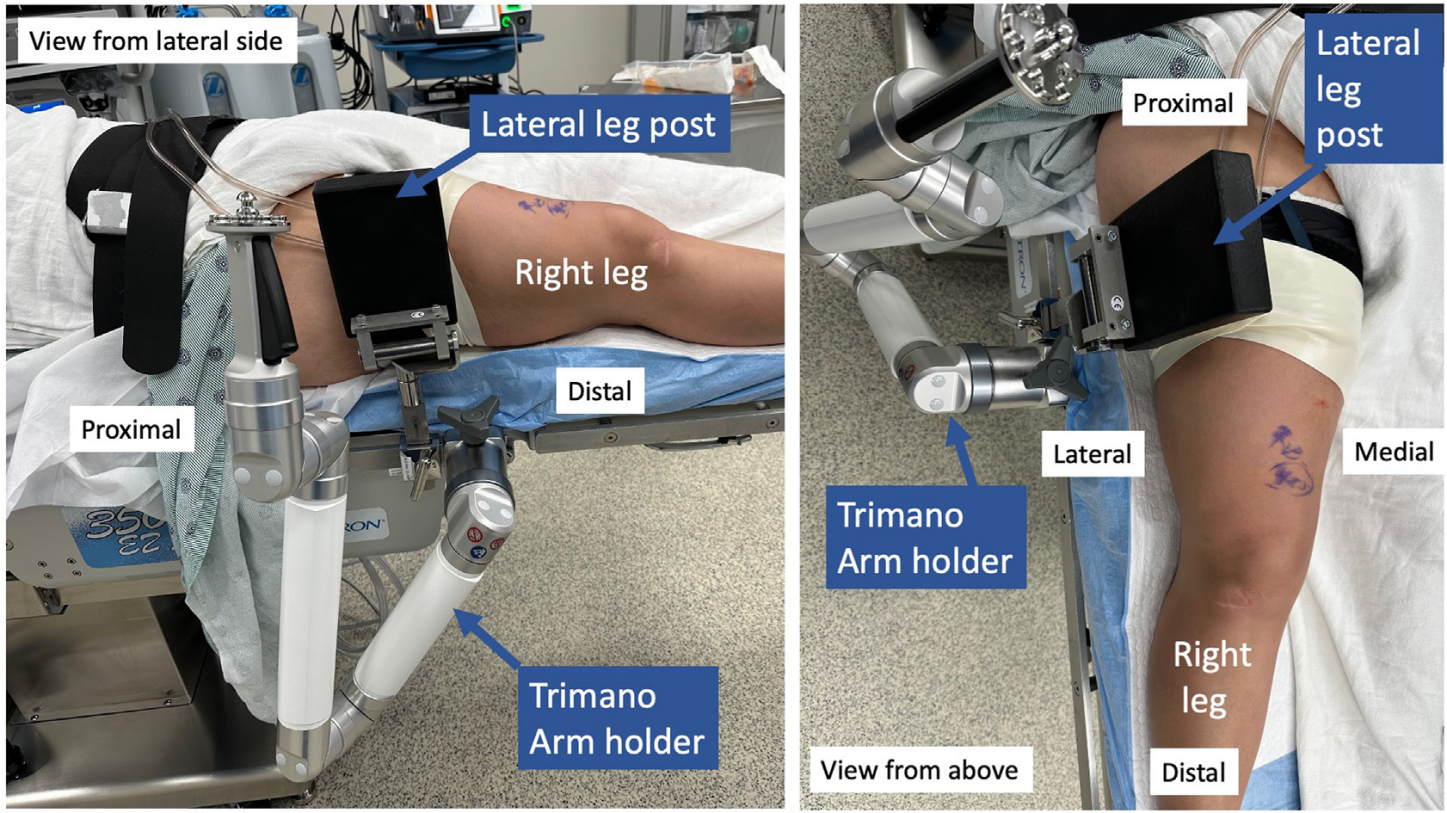
The patient is in the supine position for right knee surgery. The lateral leg post is placed at level of the tourniquet. The Arthrex TRIMANO FORTIS arm holder is placed just distal to the leg post.

After the patient is placed in the supine position and a lateral leg post is placed at the level of the tourniquet, the TRIMANO FORTIS (Arthrex, Naples, FL) arm holder is secured to the bed anterior to the lateral leg post (Video 1, Fig 1, Table 1). It can also be placed posterior to the lateral post if an increased lever arm is needed to apply a more forceful valgus stress to address medial pathology.

**Table 1:** Pearls and Pitfalls for Performing the Solo Anterior Cruciate Ligament Technique With an Articulated Arm Holder

Pearls
1. The patient is placed in the supine position without dropping the bottom of the leg.
2. A lateral leg post is required.
3. A sandbag or L-shaped holder is not needed to maintain leg in flexion.
4. The articulated arm holder can be placed anterior or posterior to the lateral leg post.
5. To address medial pathology more effectively, place the articulated arm holder posterior to lateral leg post to lengthen the lever arm for an increased valgus force.
5. Test the range of motion of the holder once secured to ensure the leg can be placed in the desired positions without any obstructions prior to draping.
6. Placing the foam piece correctly on the leg requires 2 persons.
7. Use dry arthroscopic visualization to close the quadriceps tendon.
Pitfalls
1. The sticker used to secure the sterile clear plastic TRIMANO cover to the hockey-puck connector can dislodge. Adding Coban or Ioban at the clear plastic/hockey-puck interface can be done to provide additional reinforcement.
2. The TRIMANO can get in the way of a fluoroscopy machine. Ensure appropriate fluoroscopic images can be obtained with the TRIMANO in place before draping.
3. Not being aware of the location of the round connector on the foam piece could cause iatrogenic injury to the contralateral leg in the figure-of-4 position as the connector can compress the contralateral leg.
4. The needle of the self-retrieving device can break when closing the quadriceps tendon defect distally as the tendon is thicker there. We recommend tying off the suture more proximally and switching to a regular closure with a needle driver distally.

**Fig 2:**
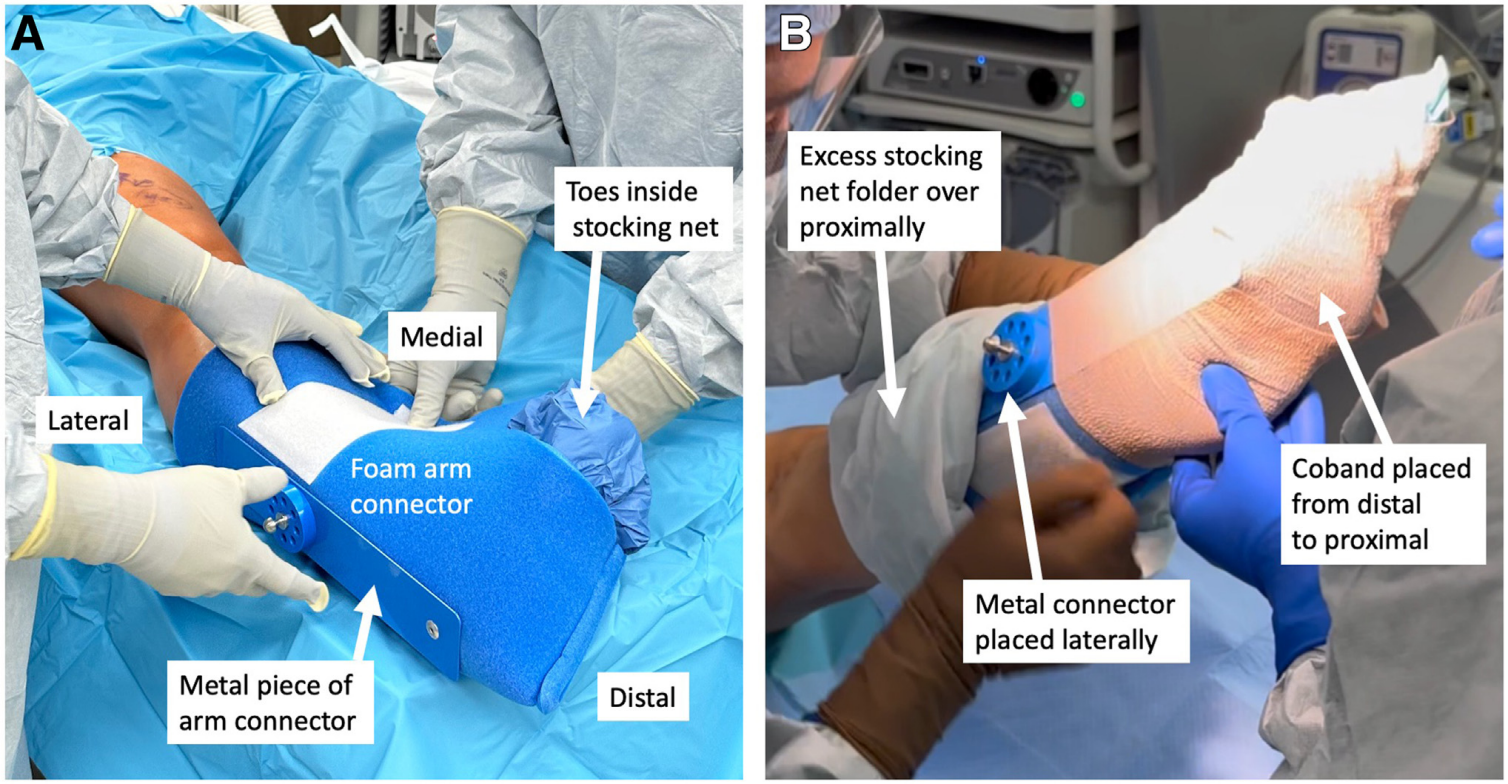
Placement of foam arm connector onto foot: In this example the patient is the supine position and the leg shown is a right leg. The foam piece is placed on the leg above the stocking net, ensuring that the outside metal piece connector is lateral and in line with the fibula. The plantar arch of the foot will rest on the cylindrical piece inside the foam connector with toes inside stocking net exposed (A). The excess stocking net is then folded over the foam proximally to expose the tibia. A Coban is then used from distal to proximal to secure the foam piece to the leg while ensuring that the metal connector piece stays lateral (B).

The leg is prepped and draped in sterile fashion. The articulated arm holder and corresponding hockey-puck connector are draped. A stocking net is placed on the leg. The corresponding TRIMANO arm foam piece is placed on the leg above the stocking net, ensuring the outside metal piece connector is lateral and in line with the fibula. The plantar arch will rest on the foam connector’s cylindrical piece (Fig 2A). The excess stocking net is folded over the foam proximally to maximally expose the tibia. Coban (3M, St. Paul, MN) is applied distal to proximal to secure the foam piece to the leg while ensuring the metal connector piece stays lateral (Fig 2B).

**Fig 3:**
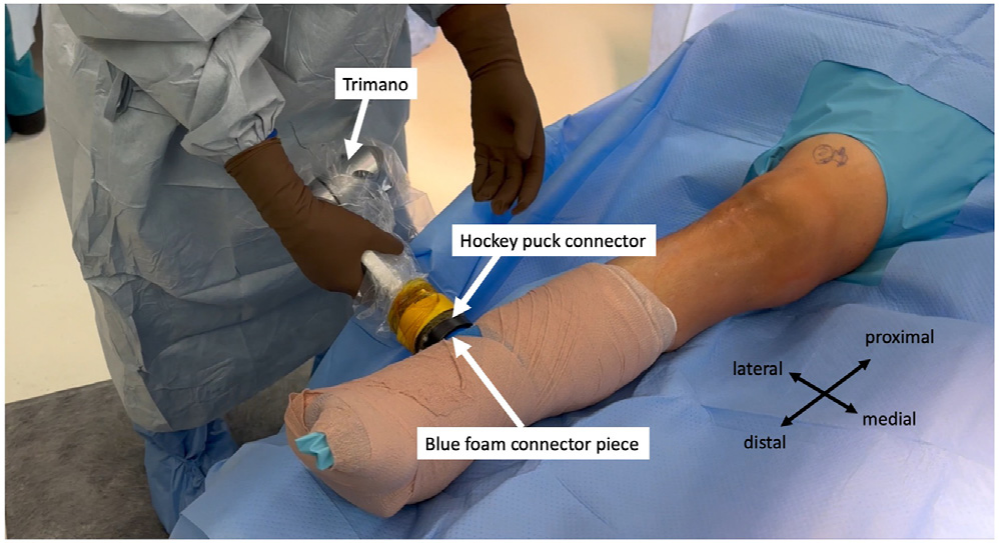
The patient is in the supine position for right leg surgery. The hockey-puck connector on the articulated arm holder is then connected to its corresponding connector on the blue foam holder.

**Fig 4:**
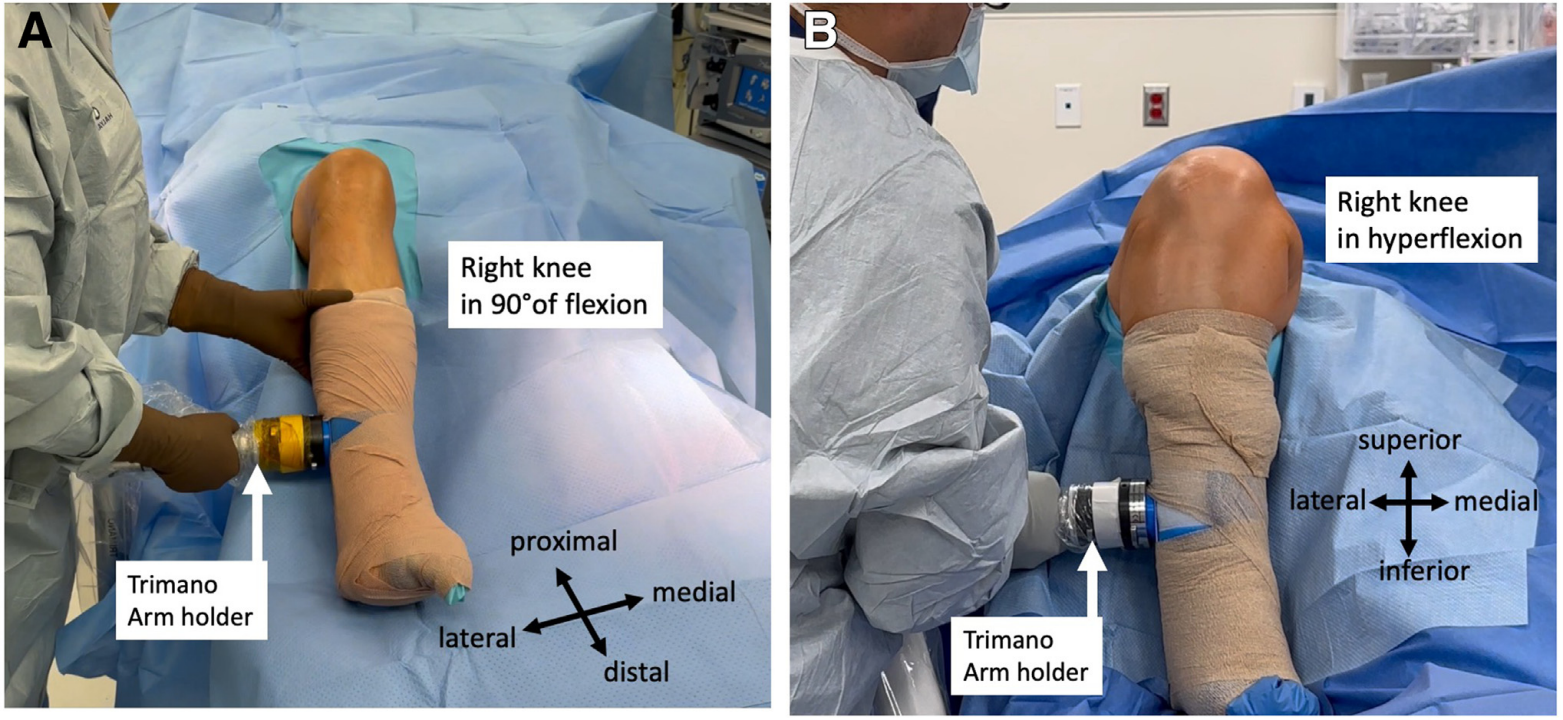
The leg range of motion with varying degrees of flexion and extension is checked. The leg can be easily placed in 90° of flexion (A) as well as hyperflexion (B).

**Fig 5:**
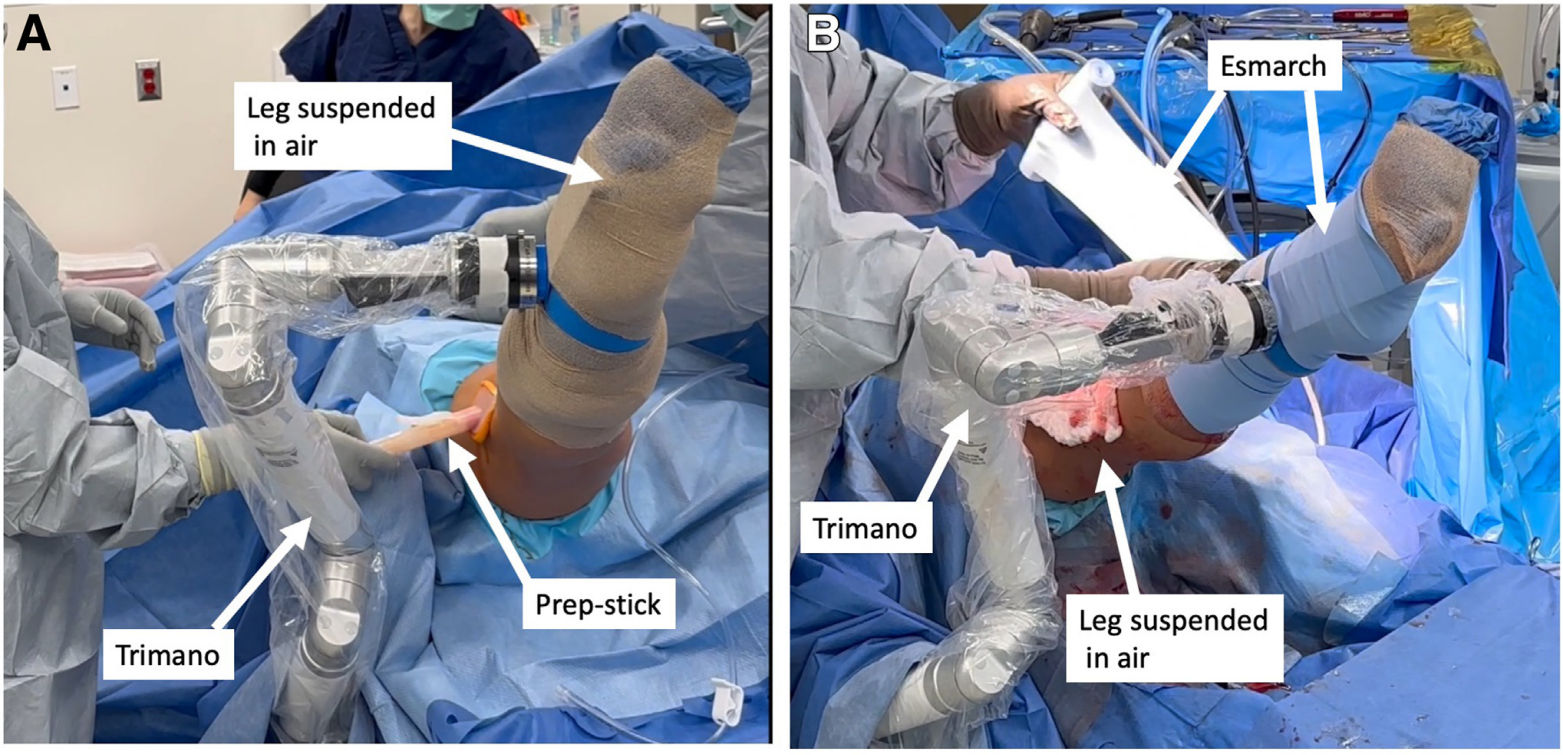
The arm holder can be used to suspend the leg in the air (right leg shown). In (A), the surgeon performs an additional prep without the use of an assist while the leg is suspended in the air. In (B), an Esmarch is used to exsanguinate the leg without assistance while the TRIMANO suspends the leg in the air.

The hockey-puck connector on the articulated arm holder is connected to its corresponding blue foam holder connector (Fig 3). The range of motion of the arm holder is verified to ensure the range of motion is uninterrupted. The leg can be placed in 90° of flexion (Fig 4A) and in hyperflexion (Fig 4B). The surgeon can use the arm holder to suspend the leg to perform additional prep (Fig 5A). The surgeon can also use an Esmarch to exsanguinate the leg without an assistant if tourniquet use is desired (Fig 5B).

Arthroscopy or graft harvest can proceed depending on the pathology or surgeon’s preference. When performing a solo ACL, we prefer performing arthroscopy first to address intra-articular pathologies before proceeding with graft harvest to minimize graft open airtime exposure.

**Fig 6:**
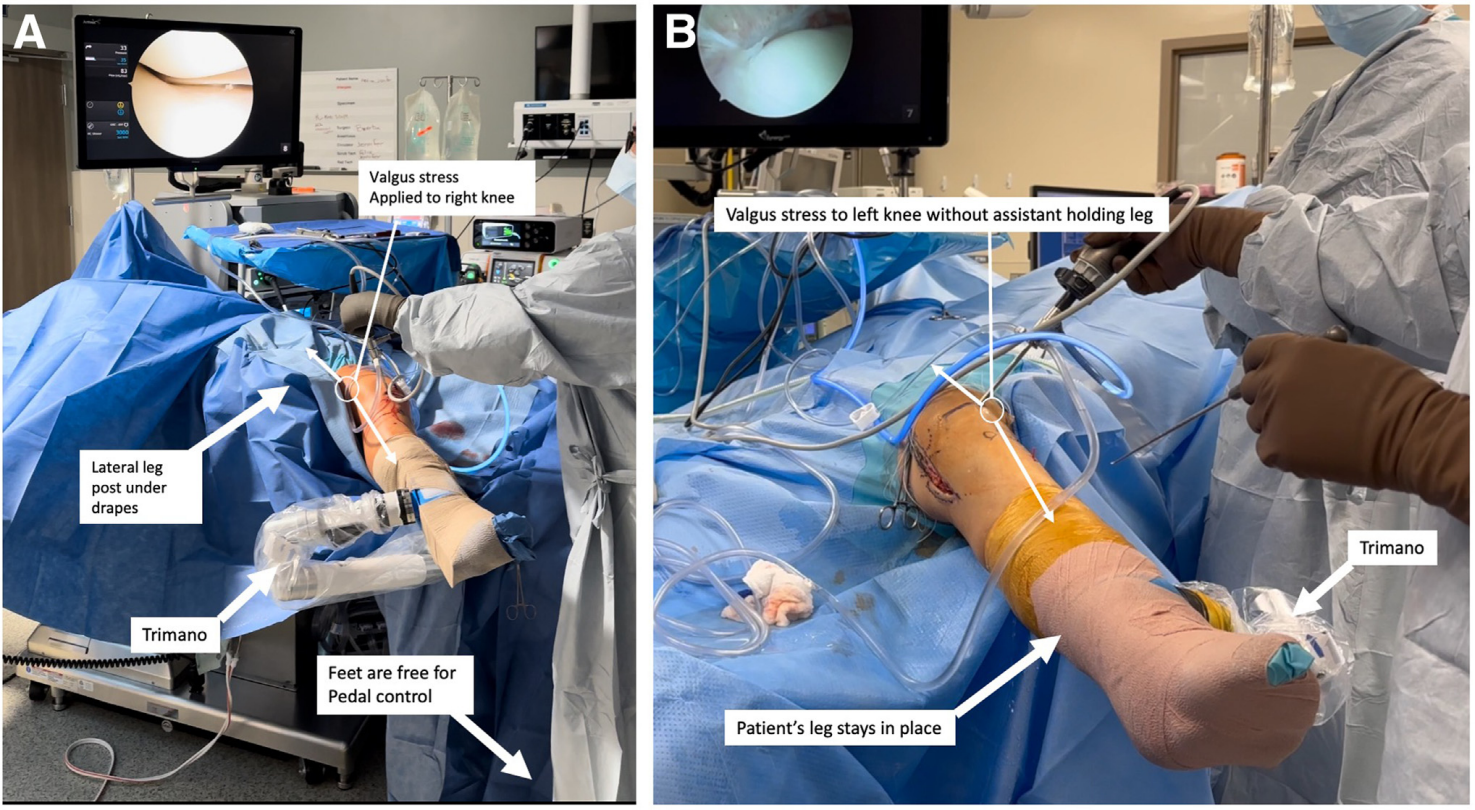
A valgus stress can be performed with the use of the lateral post and the articulated arm holder to address medial pathology. (A) In this right knee surgery example, no assistant is needed to hold the leg with a valgus stress. The holder frees the surgeon’s feet to use pedal control instead of hand control on the shaver if preferred. As shown in the left knee surgery example in (B), the leg will stay in place while a valgus stress is applied by the arm holder.

To address medial pathology, valgus stress can be applied using the lateral post and the articulated arm holder (Fig 6A). This eliminates requiring an assistant to hold the leg while applying valgus stress. As shown in Figure 6B, the leg will remain stationary. If needed, the surgeon can use his/her body for additional valgus force onto the knee. The holder also frees the surgeon’s feet for pedal control (Fig 6A).

**Fig 7:**
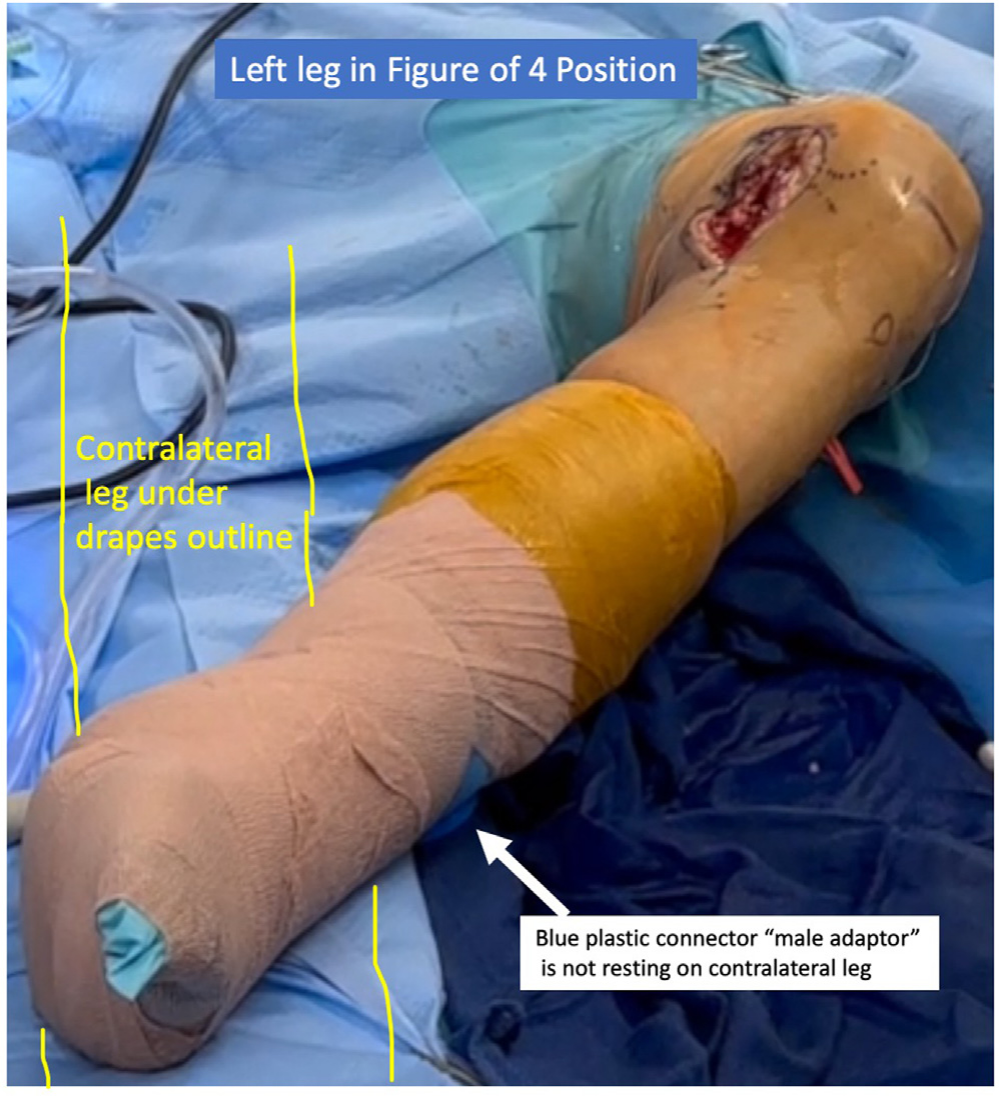
The leg is taken out of the articulated arm holder to place it in the figure-of-4 position, with care taken to ensure that the blue plastic connector (i.e., “male adaptor” part) is not resting on the patient’s contralateral leg (yellow outline) under the drapes.

To address lateral pathology, the leg is removed from the holder and oriented in the figure-of-4 position (Fig 7) while ensuring the “male” part of the lateral connector does not rest on the patient’s contralateral leg during this portion.

**Fig 8:**
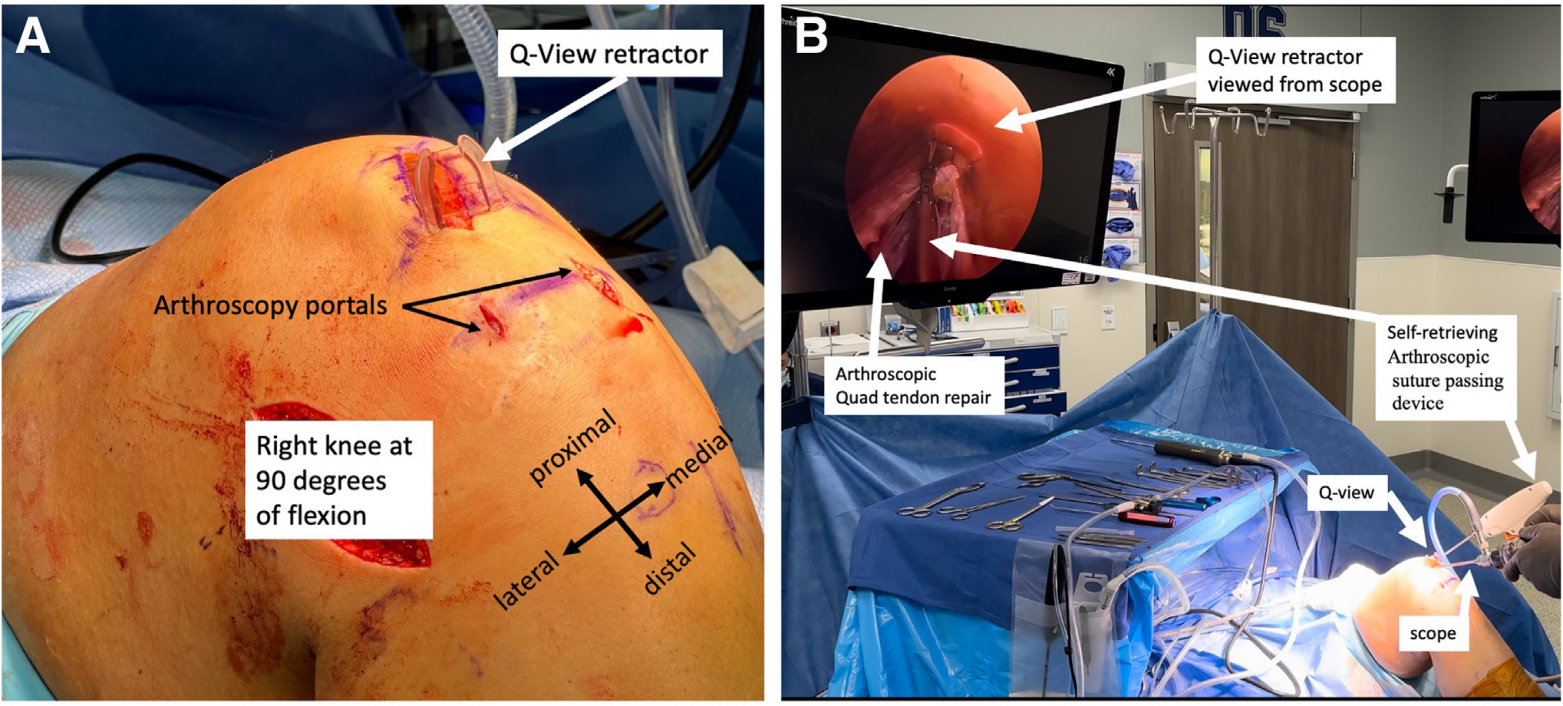
To harvest the quadriceps tendon autograft, the Smith & Nephew Q-VIEW retractor is placed inside the incision at the level of the quadriceps tendon (A, right knee). After graft harvest, the quad tendon can then be repaired without the use of an assist by using the Q-VIEW retractor, the dry arthroscope, and a self-retrieving arthroscopic suture passing device (B, left knee).

The quadriceps tendon graft harvest for ACL reconstruction can be performed without an assistant by using the Smith & Nephew (Andover, MA) Q-VIEW retractor (Fig 8A), as this retractor optimally visualizes the quadriceps tendon. Once the quadriceps tendon graft is harvested, the quadriceps tendon can be repaired without an assistant by using the Q-VIEW retractor for retraction, the dry arthroscope, and a self-retrieving arthroscopic suture-passing device (Fig 8B). The scrub technician and/or assistant can prepare a graft while the surgeon closes the quadriceps defect without requiring tissue retraction assistance.

ACL surgery can proceed after graft preparation. The articulated arm holder stabilizes the knee in hyperflexion during anteromedial portal drilling and stabilizes the leg during tibial tunnel drilling. The leg also can be held stably with the desired amount of extension while performing final graft tensioning.

## Discussion

This Technical Note proposes a solution to performing ACL surgery safely without an assistant. Although we recognize there are multiple arthroscopic setups for ACL surgery, such as dropping the leg of the table and using a well-leg holder, these setups have limitations. For example, the surgeon requires his/her body for valgus stress on the leg while operating with the dropped-leg table technique. This can be challenging for surgeons of smaller stature/size. It also requires someone to hold the leg to access the lateral compartment. Furthermore, this set-up limits pedal use, since both feet must be well planted on the floor to manipulate the leg, further challenging surgeons who prefer pedal control on arthroscopic instruments.^4^

The supine set-up with the TRIMANO has many advantages over other knee arthroscopy set-ups (Table 2). First, the TRIMANO manipulates and stabilizes the leg in any plane for an indefinite amount of time, allowing ACL surgery to be more easily performed on a leg of any size by any surgeon regardless of height by eliminating manual holding and positioning. The TRIMANO also allows for both pedal and hand control on arthroscopic instruments.

**Table 2:** Advantages/Disadvantages of the Solo ACL With an Articulated Arm Holder

Advantages
1. No surgical assist is needed to perform ACL surgery safely.
2. The knee can be placed in any degree of flexion/extension/varus/valgus without an assistant holding the leg for an indefinite amount of time.
3. If a surgical assist is available, the assist and scrub technician can prepare the graft while the surgeon performs arthroscopy and/or wound closure to optimize efficiency as no assist is needed to hold the leg.
4. An Esmarch can be used to exsanguinate the leg without anyone holding the leg allowing scrub tech to continue the surgical setup.
5. The articulated arm holder frees the surgeon to use either hand control or pedal control during arthroscopy without an assist holding the leg.
6. The articulated arm holder can support any leg size.
7. During quad tendon autograft harvest, the use of the Q-VIEW retractor and a self-retrieving suture passer allows the surgeon to close the quad defect solo.
Disadvantages
1. A lateral post is required to apply a valgus stress.
2. Greater cost: the single-use foam piece, the Q-VIEW harvest system, and the self-retrieving suture passer are all one-time use items that increase the cost of ACL surgery.
3. Doing a solo ACL will take longer than when a surgical assist available as the surgeon will have to prep the graft and close the harvest site.
4. Using the articulated arm holder for knee surgery could prevent others from using it for shoulder surgeries.

ACL, anterior cruciate ligament.

Our setup allows for ACL surgery to be performed with any type of allograft or autograft (hamstring, quadriceps tendon, bone–patellar tendon–bone). Hamstring autografts can usually be harvested, and the wound created can be closed without an assistant.^5^ Bone–patellar tendon–bone grafts can be harvested using a single scrub technician, and the wound can be closed without an assistant.^6^ Contrarily, the harvest and closure of quadriceps tendon autografts pose some disadvantages compared with other grafts because they require retraction by an assistant, making it difficult to perform solo. In addition, arthroscopy cannot be performed until the quadriceps tendon defect is closed in a watertight fashion to prevent arthroscopic fluid extravasation.^7^ The Smith & Nephew Q-VIEW retractor thus allows the surgeon to both harvest and close the quadriceps defect safely without an assistant by providing optimal visualization of the quadriceps tendon and retraction of subcutaneous tissues and skin for a solo harvest and closure. This is advantageous as the scrub technician or assistant can prepare the graft while the surgeon closes the quadriceps defect.

There are some disadvantages (Table 2) to the TRIMANO and Smith & Nephew Q-VIEW retractors, such as cost and resource availability. They both are added expenses to a surgical facility. Every TRIMANO case requires a disposable foam connector with sterilization of its corresponding hockey-puck connector. However, some facilities use generic TRIMANO foam connectors to save costs. Our technique used the Arthrex blue foam connector, so we cannot comment on the efficacy of generic foam connectors. The availability of the TRIMANO at the surgical facility may also be problematic, since surgeons use this arm holder for shoulder surgeries. Every Smith & Nephew Q-VIEW retractor case requires a plastic retractor, harvesting blades, and self-retrieving a suture-passing device. These are disposable items that add to the cost of ACL surgery.

Overall, ACL surgery, particularly when using a quadriceps tendon autograft, is a difficult operation to perform without an assistant. The use of the TRIMANO arm holder adapted as a leg holder with the Smith & Nephew Q-VIEW retractor allows a surgeon to safely perform ACL surgery with a quadriceps tendon autograft without an assistance.

## Disclosures

All authors (R.A.B., J.G., N.F.F.) declare that they have no known competing financial interests or personal relationships that could have appeared to influence the work reported in this paper.

## Supplementary Data

Video 1 Orient the patient in the supine position, place the lateral leg post at the level of the tourniquet, and secure the TRIMANO FORTIS (Arthrex) arm holder to the bed anterior to the lateral leg post. Place the TRIMANO posterior to the lateral post to address medial pathology. Prep/drape the leg in sterile fashion, drape the TRIMANO and corresponding hockey-puck connector, and place a stocking net from the lower leg to the knee. Place the corresponding TRIMANO arm foam piece on the leg above the stocking net ensuring the outside metal piece connector is lateral and in line with the fibula. Rest the plantar arch on the foam connector’s cylindrical piece. Fold excess stocking net over the foam proximally to maximally expose the tibia. Apply Coban distal to proximal to secure the foam piece to the leg while ensuring the metal connector piece stays lateral. Connect the hockey-puck connector on the articulated arm holder to its corresponding blue foam holder connector. Verify uninterrupted range of motion of the TRIMANO and ensure the leg can be placed in 90° of flexion and in hyperflexion. Perform a timeout and use an Esmarch to exsanguinate the leg if tourniquet use is desired. Arthroscopy or graft harvest can proceed depending on the pathology or surgeon’s preference. To address medial pathology, apply valgus stress using the lateral post and the TRIMANO. If needed, the surgeon can use his/her body for additional valgus force onto the knee. To address lateral pathology, remove the leg from the holder and orient it in the figure-of-4 position. Use the Smith & Nephew Q-VIEW retractor for quadriceps tendon graft harvest for ACL reconstruction. Repair the quadriceps tendon using the Q-VIEW retractor for retraction, the dry arthroscope, and a self-retrieving arthroscopic suture-passing device. A scrub technician and/or assistant can prepare a graft while the surgeon closes the quadriceps defect. Proceed with ACL surgery after graft preparation. The TRIMANO stabilizes the knee in hyperflexion during anteromedial portal drilling and stabilizes the leg during tibial tunnel drilling. The leg can also be stably held with the desired amount of extension while performing final graft tensioning.
